# PADB : Published Association Database

**DOI:** 10.1186/1471-2105-8-348

**Published:** 2007-09-19

**Authors:** Hwanseok Rhee, Jin-Sung Lee

**Affiliations:** 1 Department of Clinical Genetics, Yonsei University College of Medicine, Seoul, Korea; 2 Department of Pediatrics, Yonsei University College of Medicine, Seoul, Korea; 3 Brain Korea 21 Project for Medical Science, Yonsei University, Seoul, Korea

## Abstract

**Background:**

Although molecular pathway information and the International HapMap Project data can help biomedical researchers to investigate the aetiology of complex diseases more effectively, such information is missing or insufficient in current genetic association databases. In addition, only a few of the environmental risk factors are included as gene-environment interactions, and the risk measures of associations are not indexed in any association databases.

**Description:**

We have developed a published association database (PADB; ) that includes both the genetic associations and the environmental risk factors available in PubMed database. Each genetic risk factor is linked to a molecular pathway database and the HapMap database through human gene symbols identified in the abstracts. And the risk measures such as odds ratios or hazard ratios are extracted automatically from the abstracts when available. Thus, users can review the association data sorted by the risk measures, and genetic associations can be grouped by human genes or molecular pathways. The search results can also be saved to tab-delimited text files for further sorting or analysis. Currently, PADB indexes more than 1,500,000 PubMed abstracts that include 3442 human genes, 461 molecular pathways and about 190,000 risk measures ranging from 0.00001 to 4878.9.

**Conclusion:**

PADB is a unique online database of published associations that will serve as a novel and powerful resource for reviewing and interpreting huge association data of complex human diseases.

## Background

The importance of association databases is ever increasing and several association databases have been published. The Genetic Association Database (GAD) [1] is an archive of published genetic association data, and the Human Genome Epidemiology (HuGE) Published Literature Database (HPLD) [2] contains genetic association data published since October 2000. In addition to these general genetic association databases, other databases focus on the specific areas of genetic associations. For example, the PharmGKB database [3] specifically assembles pharmacogenetic information. The AlzGene database [4] catalogues genetic association data for Alzheimer's disease only, and the T1Dbase [5] compiles genetic association data limited to Type 1 diabetes. On the other hand, the Cancer Genetic Markers of Susceptibility (CGEMS) [6] project and the National Institute of Neurological Disorders and Stroke (NINDS) [7] maintain genome-wide association data for Parkinson's disease and prostate cancer, respectively. And the dbGaP database [8] aims to be the central repository of the genome-wide association data, although it is in its early stage. However, several important utilities are missing or insufficient in current genetic association databases. Because most genetic risk factors are believed to contribute only small influences to the development of complex diseases, molecular pathway information will prove useful to find a panel of genes that might operate together in the pathogenesis of a disease [9,10]. And it is also important that any genetic polymorphisms associated with a biological trait or disease should be carefully interpreted in the context of linkage disequilibrium (LD) between genetic markers to find a causal variant or gene. Nevertheless, molecular pathway information and the International HapMap Project [11] data cannot be effectively accessed from most of genetic association databases. Also, current 'genetic' association databases include only a few of the 'environmental' risk factors as gene-environment interactions, and no database indexes the risk measures of the associations at present.

## Construction and content

**Figure 1:**
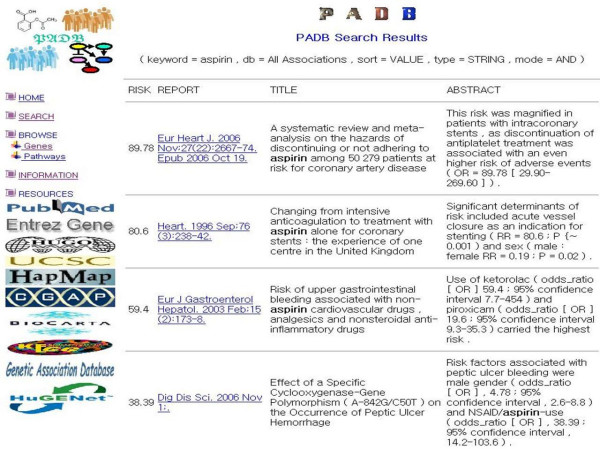
**Sorting associations by the risk measures**. PADB automatically extracts the odds ratio, hazard ratio, risk ratio and relative risk data if they are available in sentences. When multiple associations are reported in a single sentence, those multiple association data are indexed as separate records.

**Figure 2:**
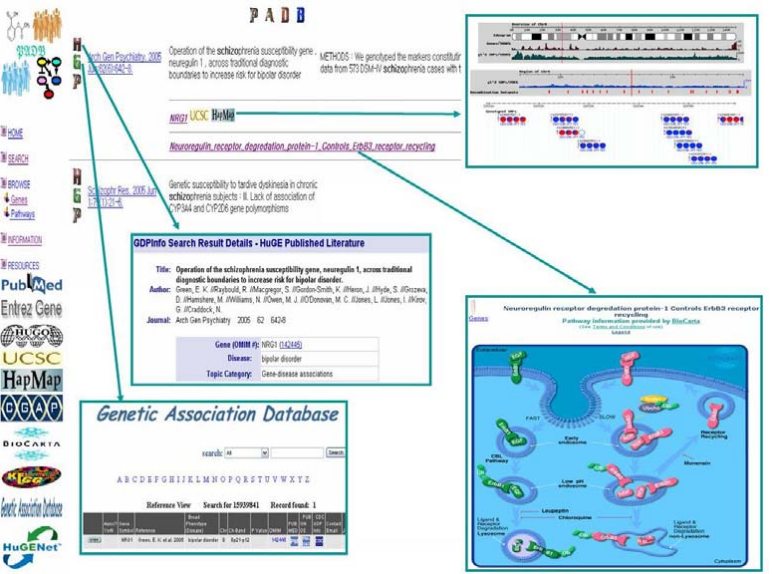
**Linking genetic risks to molecular pathway and HapMap information**. PADB can help biomedical researchers to review and interpret genetic risk factors more effectively along with molecular pathway and HapMap information.

Here we describe the development of a published association database (PADB; ), which can help biomedical researchers to review genetic and environmental risk factors more effectively along with molecular pathway and HapMap information. PADB indexes sentences containing keywords such as 'case-control', 'cohort', 'meta-analysis', 'systematic review', 'odds ratio', 'hazard ratio', 'risk ratio', 'relative risk', or 'associat*' from PubMed [12] abstracts, which are retrieved using the National Center for Biotechnology Information (NCBI) Entrez programming utilities [13]. PADB extracts the odds ratio, hazard ratio, risk ratio and relative risk measures automatically if they are available in the sentences. If multiple associations are reported in a single sentence, they are indexed as separate records (Figure 1).To expand the knowledge of genetic association data, PADB automatically identifies the HUGO official symbols of human genes [14] in the abstracts and links them to various resources such as the NCBI Entrez Gene database [15], the University of California Santa Cruz (UCSC) genome browser [16] and the International HapMap Project database. Thus, genomic annotation data including HapMap information can be assembled quickly for further analyses. If any gene participates in molecular pathways listed in the BioCarta [17] or the Kyoto Encyclopedia of Genes and Genomes (KEGG) pathway databases [18], the gene is linked to those databases through the National Cancer Institute (NCI) Cancer Genome Anatomy Project (CGAP) pathway database [19]. In addition, each record of PADB is linked to the GAD or the HPLD if it is also included in those databases (Figure 2).

## Utility and discussion

### Navigating PADB

Users can selectively search four sections of PADB, each containing genetic associations, pathway associations, any associations including the risk measures in the abstract, and all associations. The genetic associations section consists of articles containing allele-related terms such as 'allele', 'genotype', 'haplotype', 'mutation' or 'polymorphism'. If the genes are constituents of any molecular pathways listed in the BioCarta or the KEGG pathway databases, those associations can be searched in the pathway associations section. When the risk measures are reported in the abstracts, such associations can be selectively searched and sorted by the risk measures. Finally, any associations reporting either genetic or non-genetic environmental risk factors can be searched and sorted by the publication date. Because PADB searches the input text as a substring, the search for 'thyroid' retrieves articles that contain words such as 'thyroiditis', 'parathyroid' and 'hypothyroidism'. The search is case-insensitive and the search results for 'notch' and 'NOTCH' are same. Also, users can select one option on how to treat the words typed in the query text box. The words can be searched using Boolean terms such as 'AND' or 'OR'. Otherwise, the words can be searched as a phrase. For example, the search for 'mood disorder' in 'PHRASE' mode selects those sentences that contain "mood disorder in adults" but not "mood in bipolar disorder".

### Searching for environmental risk factors

The search for 'cadmium' in PADB risk measures section retrieves 55 risk measures ranging from 0.65 to 10.38, and 16 articles are retrieved in genetic associations section. The same search failed to retrieve any association from the GAD and the HPLD, because only a few environmental risk factors are included as gene-environment interactions. Various non-genetic environmental risk factors have proved strong risk factors for human diseases and most gene-environment interactions have probably not been investigated yet. Thus the search results of environmental risk factors either in the genetic associations section or in the risk measures section of PADB will provide unique and useful information.

### Sorting association data by risk measures

**Figure 3:**
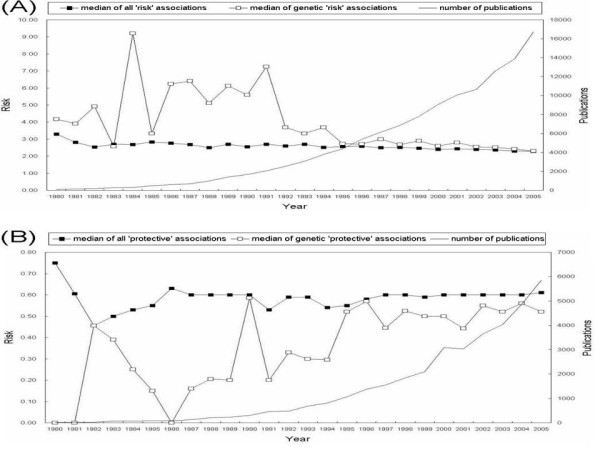
**Median risk measures of 'risk' and 'protective' associations during the past 25 years**. (A) The median strengths of risk associations remain quite stable around 2.5 (0.4 when log-transformed) in spite of the exponential increase of published association data during the past 25 years. (B) The median strengths of protective associations also remain stable around 0.6 (-0.2 when log-transformed).

Because PADB extracts the risk measure of each association, users can sort associations by risk measures. Although risk measures have become smaller on average probably thanks to improved study approaches during the past few decades (Figure 3), sorting by risk measures still might be useful to characterize or summarize the associations. For example, the search for 'cancer aspirin' in PADB risk measures section using 'AND' mode query retrieves 1088 associations reported in 284 abstracts. Among them, 94 abstracts report 208 associations, for which the risk measures range from 0.094 to 12.31. As expected, most of them show that aspirin has been associated with a reduced risk of colorectal and possibly of other common cancers. However, besides those protective associations, some strong risk associations can be identified easily, because the risk association (RR = 12.31) between aspirin use and bladder cancer mortality [20] and the raised incidence of kidney cancer (RR = 6.3) among men who took aspirin daily [21] are presented in the top rows when the search results are sorted by the risk measures. Those findings might be worth further investigation because the two risk associations are related to urological cancers. For another example, cigarette smoking is widely accepted as a major risk factor for various cancers and there are more than 2400 published risk measures, ranging from 0.05 to 435.7, in the search results for 'cancer smoking' in PADB risk measures section using 'AND' mode query. Among these, several extraordinary protective associations ranging from 0.5 to 0.9 between thyroid cancer and cigarette smoking [22-26] can be noted quickly and collected, because they are clustered in bottom rows of the search results. A similar search for '*BDNF*' retrieves 11 risk measures ranging from 1.0 to 3.81 extracted from six abstracts, and a search for '*APOE*' retrieves 435 risk measures ranging from 0.11 to 33.1 extracted from 227 abstracts. Assembling this kind of information from other databases would be very difficult.

### Expanding the scope of genetic association through pathway information

It is unlikely that any single genetic polymorphism would contribute a single critical effect to complex disease. Therefore, a pathway-based association study, which assesses a panel of polymorphisms from the genes in the same pathway, might be a good approach to find the causal genetic risk factors more effectively. For example, different genes in the same inflammatory pathway have been found to be associated with myocardial infarction [27,28], and many candidate genes of schizophrenia converge on several specific signalling networks [29-32]. Individuals with more risk alleles in the nucleotide-excision repair pathway have more elevated risks of bladder cancer [33] and several other studies also confirm the potential of applying such a pathway-based multigenic approach in association studies [34-37]. However, most genetic association studies have investigated only one or a few genes, and current genetic association databases lack sufficient molecular pathway information. Because PADB links the genetic risk factors to comprehensive molecular pathway information listed in the BioCarta and KEGG databases, it will provide novel and powerful clues for expanding the knowledge of genetic associations. For example, the search for the string 'schizo' in PADB pathway associations section retrieves numerous pathways related to various genes, including *NRG1 *[38] and *EGF *[39]. *NRG1 *is listed in the BioCarta database as *NDF *in 'h_ErbB3Pathway' where the neuregulin receptor degradation protein 1 controls *ERBB3 *receptor recycling. Because *EGF *is listed in the same pathway, the results of independent studies that report the association of *NRG1 *and *EGF *with schizophrenia can be interpreted based on the convergence of related molecular pathways. Other genes in the same pathways where *NRG1 *or *EGF *participates might also be good candidates for further investigation of genetic association with schizophrenia. On the other hand, 'MAPK signaling pathway' shared by *AKT1 *and *EGF *could be also a good candidate pathway for further studies. Interestingly, *ERBB3 *gene participates in both 'h_ErbB3Pathway' and 'MAPK signaling pathway'. Thus, these evidences might imply an important role of *ERBB3 *gene in the pathogenesis of schizophrenia. Because the links are established through automatically identified human gene symbols, the article "Association of smoking, CpG island methylator phenotype and V600E *BRAF *mutations in colon cancer" is linked to various pathways, including the 'Alzheimer disease pathway' in which the *BRAF *gene is involved. Since more than 45 articles can be found by the search for 'alzheimer colon cancer' in PubMed, the connection between 'Alzheimer disease pathway' and 'colon cancer' through *BRAF *gene might imply novel mechanism shared by the two diseases.

### Expanding the region of genetic association through HapMap information

Any associated genetic markers might be causal alleles or just strongly linked with the causal allele. For example, several single nucleotide polymorphisms (SNPs) in *IFIH1 *region show association with Type 1 diabetes. However, the associated region contains four genes and there are strong LDs between SNPs across this region. Thus, we need additional data to determine which of the four genes is likely to be the causal locus [40]. Although many genetic association studies have focussed on non-synonymous SNPs, the causal alleles of complex diseases are far less likely to be missense variations [41]. Indeed, the Thr17Ala polymorphism in *CTLA4 *is associated with autoimmune disease only because it is in strong LD with a regulatory polymorphism that is more strongly associated with disease and is therefore more likely to be the causal allele [42]. Because the HapMap data include the extent of LD across the genome and the minor allele frequencies of SNPs measured from four ethnic groups, the target region of any genetic association can be expanded based on LD. For example, *ACSL6 *gene was reported to be associated with schizophrenia [43,44], and these associations can be retrieved in the search results for 'schizophrenia *ACSL6' *in PADB genetic associations section using 'AND' mode query. The HapMap link of the gene shows that there is strong LD across a 200 kb genomic region around *ACSL6 *gene. Thus, PADB users can easily select several genes such as *IL3 *or *CSF2*, which are good candidates for further genetic association studies due to strong LD with *ACSL6*. In fact, multiple SNPs located in and around *IL3 *gene were recently found to be associated with schizophrenia [45].

### Comparison with PubMed database

The results of association studies have been reported using different risk values such as odds ratios, relative risks, hazard ratios or risk ratios, according to the design and analysis method of the study, and each of them is expressed exclusively against other measures. Accordingly, the search results of various keywords related to association studies in PubMed rarely overlap (Table 1). For example, the search for 'odds ratio*' in PubMed retrieved 67,815 abstracts (accessed on 30 Nov. 2006) and among them 65,047 (95.9%), 66,998 (98.7%) and 67,238 (99.2%) abstracts did not contain 'relative risk*', 'hazard ratio*' or 'risk ratio*', respectively; in addition, 50,804 (74.9%) and 56,612 (83.5%) abstracts did not contain 'case-control' or 'cohort*', respectively. By comparison, 75,253 (81.6%) abstracts out of 92,264 containing 'case-control' and 117,925 (91.3%) abstracts out of 129,128 containing 'cohort*' did not contain 'odds ratio*'. Thus, it would be a very error-prone task for researchers to capture all of the relevant articles from PubMed using such a long and complex query process without the aid of an association database such as PADB.

**Table 1:** PubMed search results of various query terms related to association studies

Initial Query	Results	Combined Query Results						
		
		NOT (odds ratio*)	NOT (relative risk*)	NOT (hazard ratio*)	NOT (risk ratio*)	NOT (case-control)	NOT (cohort*)	NOT (associa*)
odds ratio*	67,815	-	65,047	66,898	67,238	50,804	56,612	24,427
relative risk*	32,280	29,512	-	31,944	31,830	27,878	24,374	14,907
hazard ratio*	8,927	8,110	8,591	-	8,868	8,598	5,848	3,548
risk ratio*	3,694	3,117	3,244	3,535	-	3,377	2,814	1,841
case-control	92,264	75,253	87,862	91,935	91,947	-	83,109	50,155
cohort*	129,128	117,925	121,222	126,049	128,248	119,973	-	75,875
associa*	1,591,541	1,548,153	1,574,168	1,586,162	1,589,688	1,549,432	1,538,288	-

The search results for various keywords related to association studies in PubMed seldom overlap. The search for 'odds ratio*' in PubMed retrieved 67,815 abstracts when accessed on 30 November 2006. Among these, 65,047 (95.9%), 66,998 (98.7%) and 67,238 (99.2%) abstracts did not contain 'relative risk*', 'hazard ratio*' or 'risk ratio*', respectively. Moreover, 50,804 (74.9%) and 56,612 (83.5%) abstracts did not contain 'case-control' or 'cohort*', respectively. By comparison, 75,253 (81.6%) abstracts out of 92,264 containing 'case-control' and 117,925 (91.3%) abstracts out of 129,128 containing 'cohort*' did not contain 'odds ratio*'.

### Comparison with other association databases

The GAD and the HPLD contain only a few environmental risk factors as gene-environment interactions, as discussed above. Furthermore, the pathway database links provided by the GAD seldom work and the HPLD does not provide any links to the pathway databases. Because the HPLD uses a controlled vocabulary, the search results for 'stroke' successfully include related terms such as 'cerebrovascular disease' and 'transient ischemic attack'. However, the users cannot search for terms such as 'rs4680' or 'Chinese' in the abstract because they are not included in the standard vocabulary. The PharmGKB database specifically focuses on the pharmacogenetic association data only and also lacks environmental factors. The Cochrane Database of Systematic Reviews offers only systematic reviews, and genetic associations are seldom reviewed. Although the AlzGene database and the T1Dbase have many excellent features, including meta-analysis or microarray data, each database covers only one specific disease. Recently, genome-wide association databases such as CGEMS, NINDS and dbGaP have been introduced. Because these databases store the results of genome-wide association studies on a specific disease, they also differ from general association databases such as PADB, GAD and HPLD (Table 2).

**Table 2:** Comparison of association databases

	Data coverage			Search type		Special content		Data presentation		
	
Database	genetic risk factors	environmental risk factors	all research area	free text	controlled vocabulary	sample size information	systematic analysis results	sorting by risk measures	link to HapMap database	link to pathway database
PADB	O	O	O	O	X	X	X	O	O	O
GAD	O	Partial	O	O	X	Partial	X	X	O	O
HPLD	O	Partial	O	Partial	O	Partial	X	X	X	X
Cochrane	Partial	O	O	O	X	O	O	X	X	X
AlzGene	O	X	X	O	X	O	O	X	X	X
T1DBase	O	X	X	O	X	X	X	X	X	O
PharmGKB	O	X	X	O	Partial	X	X	X	X	O
CGEMS	O	X	X	X	X	O	O	X	X	X
NINDS	O	X	X	X	X	O	O	X	X	X
dbGaP	O	X	X	X	X	O	O	X	X	X

PADB was compared with other association databases, including those for general associations (GAD, HPLD and the Cochrane Reviews), genetic associations for specific diseases (AlzGene, T1Dbase and PharmGKB) and genome-wide associations (CGEMS, NINDS and dbGaP).

## Conclusion

PADB is a unique online database of published association data. As it automatically collects and updates association data directly from PubMed database, it is comprehensive and up-to-date. PADB covers both genetic and environmental risk factors, along with molecular pathway and HapMap information. PADB will thus serve as a novel and powerful resource for reviewing and interpreting disease association data.

## Availability and requirements

Project name: Published Association Database

Project home page: 

Operating system: Linux

Programming language: Perl and Pascal

Licence: the database is freely accessible for academic users under the GNU GPL.

Restrictions to use by non-academics: commercial users are referred to the developers of PubMed, Entrez Gene, CGAP Gene, UCSC Genome Browser, HapMap Project, BioCarta, KEGG, GAD and HPLD databases for more details on access.

## Authors' contributions

HR developed the database and takes responsibility for the integrity of the data and the accuracy of the data analyses. JL participated in the design of the database and helped to draft the manuscript. All authors read and approved the final manuscript.
